# The relationship between physiological and mechanical properties of *Acer platanoides* L. and *Tilia cordata* Mill. leaves and their seasonal senescence

**DOI:** 10.1038/s41598-019-40645-z

**Published:** 2019-03-12

**Authors:** Anna Ciupak, Agata Dziwulska-Hunek, Bożena Gładyszewska, Anita Kwaśniewska

**Affiliations:** 1 Department of Physics, University of Life Sciences, Akademicka 13, 20-950 Lublin, Poland; 2 Department of Applied Physics, Lublin University of Technology, Nadbystrzycka 38 D, 20-618 Lublin, Poland

## Abstract

The seasonal senescence of leaves in the phenological cycle coincides with the change of their strength properties which determine resistance to environmental conditions and the efficiency of the photosynthesis process. That affects the development, growth and condition of the plant. Therefore, the aim of this paper was to observe and compare the results of strength tests performed on the leaves of two species of trees popular in Poland - lime and maple. As well as chlorophyll fluorescence and photosynthetic pigments content in the context of the changes occurring during the entire leaf life cycle. Obtained results showed that the strength properties of the tested leaves reached the minimum values in spring and the maximum in the summer similarly to the leaf greenness index. Whereas the fluorescence increased which the seasonal senescence in opposition to the photosynthesis efficiency of the leaves. Collected data revealed that strength parameters and photosynthetic pigment content were significantly higher for maple leaves than for lime leaves. Studies showed differences between physiological and mechanical properties of the leaves of two trees species, even if they grew under the same environmental conditions. It is concluded from the results that phenotype and physical parameters of leaves are related to seasonal senescence.

## Introduction

The main factor initiating the appearance of the first leaves on trees is appropriate temperature in spring as well as its changes resulting from climate warming^1,2^. Leaf senescence is not only the final stage of their development but also a programmed plan for nutrient relocation within the plant^3,4^. Quantitatively, it is expressed through a number of molecular, morphological and physiological parameters^5–8^, moreover, chlorophyll fluorescence measurements are becoming increasingly popular^9–11^. For this reason change of photosynthetic pigment content (chlorophylls and carotenoids), chlorophyll fluorescence, quantum efficiency of photochemical reaction (Y (II)), the rate of electron transport through photosystems (ETR) and the leaf greenness index of the maple and lime were examined due to their life cycle, which in Poland lasts for three seasons. These phenotype parameters refer to whole plant growth traits and its productivity in quantitative and qualitative terms.

However, above- mentioned phenotype parameters are seldom related to the changes of the mechanical properties that occur throughout leaf life cycle. The importance of knowing the strength properties of these delicate biological materials can be recognized by observing the growth of the leaf under controlled external load^12^, the subsequent intended use of the leaves^13–17^, the load resistance of its anatomical parts^18–20^, the decomposition rate of leaf litter^21,22^, damage mitigation^23,24^, pest management^24–32^ and other abiotic factors^23,24^ such as wind^33,34^ and drought^20,35,36^, as well as the adaptation of plants to grow in hot dry climates^37–39^.

The complex internal structure of plant material sometimes makes it difficult to describe the mechanical properties by means of the physical quantities known from materials engineering^40–43^. As far as plant leaves are concerned, the strength properties are determined on the basis of three main groups of mechanical tests - the shear test, puncture test, and tensile test^13,16,26,28,29,32,34,37,38,44^.

In the part concerning the mechanical properties of the leaves of two species, namely - the maple and lime tree^45,46^, the authors of the paper have focused on defining selected strength parameters on the basis of the single-axial tensile test. The Young’s modulus *E* which indicates the resistance of the leaf tissue to elastic deformation (stiffness). The critical surface tension σ_*k*_ expresses the stress value at which a sample fractures. Whereas the force to tear *f*_*t*_ denotes the mechanical resistance to a pulling apart force and Poisson’s ratio *v* shows the way the material tend to deformation. These leaf mechanical traits contribute significantly to defense from insect herbivores^31,32^ and resistance to habitat factors^33^ as well as the use of leaves as a source of biomass^21,22^. The tensile test is particularly important in the case of heterogenic^14^ and different thin-film^47–50^ plant materials as it provides valuable information about their mechanical properties also in the fiber-specific direction^15,17^. Furthermore, for plant materials it is difficult to determine the value of Poisson’s ratio that is the reason for the rare testing the strength of biomaterials^51–54^. Therefore, by setting this important parameter for strength, we used our innovative authorial method of random markers^47^. On the basis of this method, the authors determined the value of the above mentioned quotient for the tree leaves under examination. Moreover, we would like to emphasize the usability and applicability of our method for testing various thin-film materials.

In spite of numerous reports determining physiological, mechanical or biochemical properties of plant materials there is still a lack of data investigating correlations between these parameters during the leaf lifespan. In the light of our findings, changes in phenotype and physical parameters in the leaf life cycle arise from the seasonal senescence. For this reason, in our opinion, future work should focus on leaf structure, as it is the most important parameter that affect in macro-scale toughness^41,52^ which vary seasonally as its chemical components change^55^.

## Results

Statistical analysis of the strength test results was performed by means of the StatSoft Inc. STATISTICA ver. 13.1 program^56^. However, due to the fact that in individual cases the assumptions required for the use of parametric statistical tests were not fulfilled by the measurable variables, the analysis of the obtained test results was performed on the basis of non-parametric tests. For analyzing the differences in the value of Young’s modulus *E*, critical surface tension *σ*_*k*_, Poisson’s ratio *v* and the force to tear *f*_*t*_ of the leaves of the examined tree species, and for determining the differences between values such as *E*, *σ*_*k*_, *v* and *f*_*t*_ which occurred as a result of the time of the year when the measurements were conducted (May-spring, August-summer, October-autumn), the ANOVA signed rank Kruskal-Wallis test and the median test were used, followed by multiple comparisons of average ranks for all the assays at materiality level α = 0.05^57^. The results of studies on the photosynthetic pigment content, chlorophyll fluorescence, quantum efficiency of photosynthesis Y(II), electron transport rates ETR and the leaf greenness index were statistically analyzed by ANOVA, by means of the NIR test and the application of STATISTICA ver. 13.1 software. The above test parameters were compared between the tree species and the seasons.

**Figure 1:**
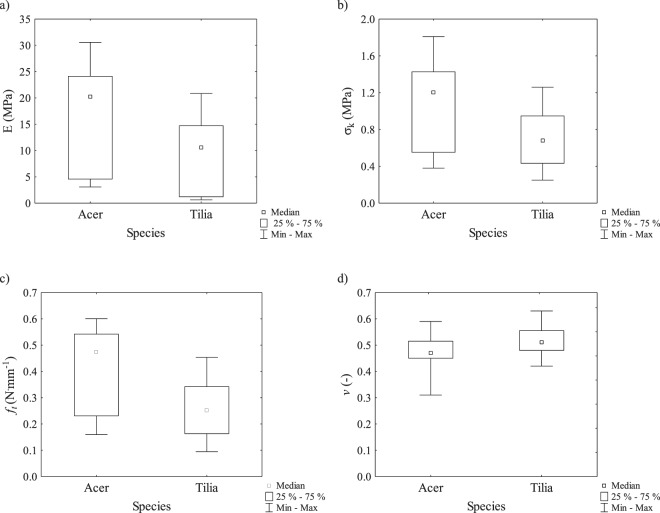
Relationship between Young’s modulus *E* (**a**), critical surface tension *σ*_*k*_ (**b**), the force to tear *f*_*t*_ (**c**), and Poisson’s ratio *v* (**d**) in relation to the tree species.

**Figure 2:**
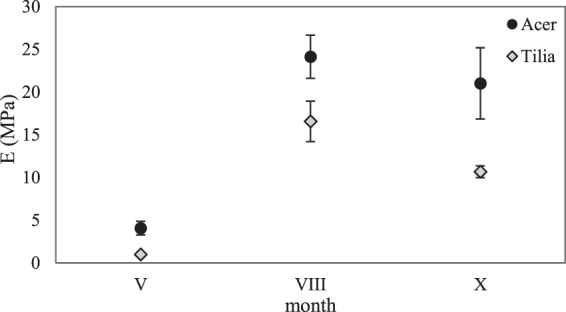
Average values of Young’s modulus *E* (MPa) of *Acer platanoides* L. maple leaves and *Tilia cordata* Mill. lime leaves with standard deviation, determined in consecutive seasons of the year; V - May (spring), VIII - August (summer), X - October (autumn).

**Figure 3:**
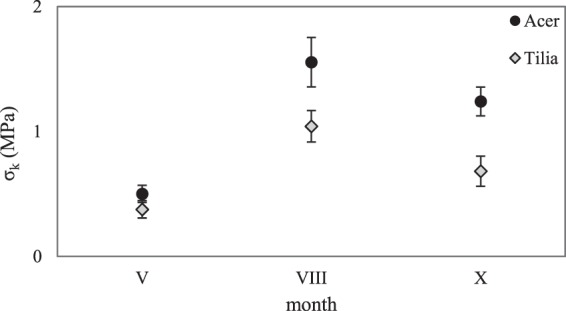
Average values of critical surface tension *σ*_*k*_ (MPa) of *Acer platanoides* L. maple leaves and *Tilia cordata* Mill. lime leaves with standard deviation, determined in consecutive seasons of the year; V - May(spring), VIII - August (summer), X - October (autumn).

Tables 1 and 2 show the average values of Young’s modulus *E*, critical surface tension *σ*_*k*_, Poisson’s ratio *v*, the force to tear *f*_*t*_ (Table 1) and the median (Table 2) with a standard deviation, determined for the leaves of the examined tree species. Statistical analysis revealed that at materiality level α = 0.05 there occur substantial differences in the values of all tested leaf strength parameters, depending on the tree species (Fig. 1). In the case of maple leaves, the values of Young’s modulus *E*, critical surface tension *σ*_*k*_ and the force to tear *f*_*t*_ were higher than those of lime leaves (Fig. 1a–c), for which only Poisson’s ratio was higher than that of maple leaves (Fig. 1d). In addition, a constant upward tendency of the strength parameters under examination was observed, starting from spring (minimum) to their maximum value in summer to be then lowered in autumn (Figs 2, 3 and Table 2).

**Table 1:** Average values of Young’s modulus *E*, critical surface tension *σ*_*k*_, Poisson’s ratio *v*, and the force to tear *f*_*t*_ of maple leaves *Acer platanoides* L. and lime leaves *Tilia cordata* Mill., determined in consecutive seasons of the year; V - May (spring), VIII - August (summer), X - October (autumn).

Month	*E* (MPa)		*σ*_*k*_ (MPa)	
	Maple (*Acer*)	Lime (*Tilia*)	Maple	Lime
V	4.06 ± 0.80^a^	1.01 ± 0.25^a^	0.50 ± 0.07^a^	0.38 ± 0.07^a^
VIII	24.13 ± 2.53^b^	16.57 ± 2.37^c^	1.56 ± 0.20^c^	1.04 ± 0.13^c^
X	21.01 ± 4.17^b^	10.67 ± 0.69^b^	1.24 ± 0.12^b^	0.68 ± 0.12^b^
	***v*** **(−)**		***f***_***t***_ (**N** · **mm**^**−1**^)	
	**Maple**	**Lime**	**Maple**	**Lime**
V	0.44 ± 0.05^a^	0.52 ± 0.05^a^	0.21 ± 0.03^a^	0.14 ± 0.05^a^
VIII	0.49 ± 0.04^b^	0.51 ± 0.06^a^	0.50 ± 0.03^b^	0.38 ± 0.05^c^
X	0.49 ± 0.03^b^	0.51 ± 0.04^a^	0.52 ± 0.06^b^	0.25 ± 0.04^b^

Values shown are the means ± SE.

^a–c^Values marked with different letters (in columns) indicate significant differences at p ≤ 0.05.

**Table 2:** Median value of Young’s modulus *E*, critical surface tension *σ*_*k*_, Poisson’s ratio *v* and the force to tear *f*_*t*_ of maple leaves *Acer platanoides* L. and lime leaves *Tilia cordata* Mill. determined in consecutive seasons of the year; V - May (spring), VIII - August (summer), X - October (autumn).

Month	*E* (MPa)		*σ*_*k*_ (MPa)		*v* (−)		*f*_*t*_ (N · mm^−1^)	
	Maple (*Acer*)	Lime (*Tilia*)	Maple	Lime	Maple	Lime	Maple	Lime
V	3.98^a^	1.03^a^	0.51^a^	0.37^a^	0.44^a^	0.53^a^	0.21^a^	0.14^a^
VIII	24.17^b^	15.82^c^	1.59^c^	1.02^c^	0.48^b^	0.52^a^	0.51^b^	0.37^c^
X	21.33^b^	10.62^b^	1.24^b^	0.68^b^	0.48^b^	0.50^a^	0.52^b^	0.25^b^

^a–c^Values marked with different letters (in columns) indicate significant differences at p ≤ 0.05.

For maple leaves the lowest *E* value (about 4 MPa) was obtained in spring, while the highest one in summer - over 24 MPa. Comparable values of this strength parameter (21–24 MPa) and no statistically significant differences were obtained for the leaves of this tree species tested in summer and autumn (Tables 1 and 2). As a result of multiple comparisons of mean ranks for all the trials at materiality level α = 0.05, statistically significant differences in Young’s modulus of lime leaves were obtained, depending on the season. Just as in the case of maple leaves, the lowest *E* value of lime leaves (approx. 1 MPa) was determined in spring. In summer, the *E* value reached the maximum of approx. 16.5 MPa, while in autumn it decreased by over 35% (to approx. 10 MPa) (Fig. 2).

A thorough analysis of the results showed a difference in critical surface tension *σ*_*k*_ of the leaves of both tree species, depending on the seasons. The *σ*_*k*_ value was lowest in spring and it approximated 0.5 MPa in the case of maple leaves and 0.38 MPa for lime leaves. The highest *σ*_*k*_ value was determined in summer and it was approximately 3 times higher than the value obtained in spring (Fig. 3, Table 2). At the end of the growing season of trees (autumn), a significant decrease in the value of critical surface tension was observed in comparison to the result obtained in summer. The value approximated 22% in the case of maple leaves and 33% for lime leaves (Tables 1 and 2).

The force to tear *f*_*t*_ of the maple leaf samples was higher than that of the lime leaves (Fig. 1c). However, statistically significant differences in *f*_*t*_ values for the maple tree were found only between the leaves tested in spring, where the value of this parameter was the lowest and amounted to 0.21 N · mm^−1^, and those obtained in summer or autumn. For those groups no statistically significant differences in *f*_*t*_ values were observed and they ranged from 0.5 N · mm^−1^ to 0.52 N · mm^−1^ (Tables 1 and 2). The season of the year affected the variation in *f*_*t*_ values of lime leaves. The lowest value was determined in spring (0.14 N · mm^−1^). In summer it was over 2.5 times higher than in spring and in autumn it decreased to approx. 0.25 N · mm^−1^ (Tables 1 and 2).

As for Poisson’s ratio *v* of lime leaves, the season of the year did not significantly affect the variety of its value, which was at a comparable level (Tables 1 and 2). For maple leaves the *v* quotient was statistically different only between the leaf groups examined in spring and summer. For autumn and summer leaves, the *v* value was determined at the same level (Tables 1 and 2).

Table 3 depicts the variation of the photosynthetic pigment content determined by the spectrophotometric method, and the leaf greenness index defined by means of SPAD-502 with regard to the seasons. Both pigment concentration (chlorophyll *a* and *b*, carotenoids) and the leaf greenness index were different for both tree species and the date of the measurement. It is obvious that the greenness index varies in relation to the season. The lowest value was obtained in autumn for lime leaves, while the highest one in spring for both tree species. The leaf greenness index determined in summer amounted to 40.81 for the lime tree and 42.36 for the maple tree. A small chlorophyll amount and nearly 5 times higher carotenoid concentration in the autumnal yellow maple leaves precluded the determination of this index by means of SPAD-502 (Table 3).

**Table 3:** Photosynthetic pigment content (chlorophyll *a* and *b*, carotenoids) determined by means of the spectrophotometric method and the greenness index determined by means of the chlorophyll SPAD-502 meter of lime and maple leaves in relation to the seasons of the year.

The tree species	The season of the year	The greenness index (unit SPAD)	Chl *a* (µg · g^−1^)	Chl *b* (µg · g^−1^)	Car (µg · g^−1^)
Lime (*Tilia*)	spring	29.97^a^	587.82^a^	134.01^a^	147.62^a^
Maple (*Acer*)	spring	32.24^b^	2062.40^b^	779.73^b^	205.55^a^
Lime	summer	40.81^c^	397.67^a^	141.36^a^	168.87^a^
Maple	summer	42.36^c^	2444.50^c^	897.58^c^	341.93^b^
Lime	autumn	12.76^d^	102.37^d^	63.52^a^	165.23^a^
Maple	autumn	—	77.95^d^	90.89^a^	383.99^b^

^a–c^Values marked with different letters (in columns) indicate significant differences at p ≤ 0.05.

Moreover, a significant reduction in chlorophyll *a* concentration in maple leaves was observed in autumn. The maximum value determined in summer (2444.5 μg · g^−1^) was over 30 times higher than the minimum one obtained in autumn (approx. 78 μg · g^−1^). The highest content of this pigment in lime leaves (approx. 588 μg · g^−1^) was determined in spring. In autumn it decreased by a factor of five (Table 3). The results of the study also indicate an increase in chlorophyll *b* content in the leaves examined in summer as compared to spring, and its significant decrease in autumn. Distinct differences in chlorophyll *b* content in different seasons of the year were noticed only in the case of maple leaves, whereas the changes in the content of this pigment were not statistically significant as far as lime leaves are concerned (Table 3).

Table 3 also shows the content of carotenoids, i.e. other photosynthetic pigments found in leaf chloroplasts. In this case, no significant differences in the amount of these dyes in the leaves under examination were observed. The concentration of carotenoids remained at a comparable level as far as both the tree species and the seasons are concerned (Table 3).

Table 4 shows the results of fluorescence measurement F/Fm′, the efficiency of photosynthesis Y(II) and electron transport rates through photosystems ETR. The results differed both in terms of tree species and the seasons. Moreover, it was noted that depending on the time of the year, both the photochemical quantum efficiency of Photosystem II and electron transport efficiency ETR varied. A low ETR value of 0.35 for autumn leaves of the maple tree was indicative of their poor photosynthetic activity. In this case, chlorophyll *a* content was also very low and amounted to approx. 78 μg · g^−1^ (Table 3), which indicated a decrease in the quantum efficiency of photochemical reaction closely related to chlorophyll content. The reduced electron transport rate ETR in autumn also signified a deterioration in the physiological state of the leaves under examination as a result of the ongoing senescing process.

**Table 4:** Quantum efficiency of photochemical reaction in PSII and ETR electron transport rate in lime and maple leaves in relation to the seasons of the year.

The tree species	The season of the year	F/Fm′	Y (II)	ETR
Lime (*Tilia*)	spring	0.337^a^	0.663^a^	6.17^a^
Maple (*Acer*)	spring	0.285^b^	0.715^b^	2.27^a^
Lime	summer	0.413^c^	0.587^c^	53.56^b^
Maple	summer	0.331^a^	0.669^a^	18.97^c^
Lime	autumn	0.572^d^	0.428^d^	5.96^a^
Maple	autumn	0.674^e^	0.326^e^	0.35^a^

^a–c^Values marked with different letters (in columns) indicate significant differences at p ≤ 0.05. F/Fm′ - min/max fluorescence of the sample adapted to light; Y(II) - quantum efficiency of photochemical reaction in PSII, value approximating the efficiency of photosynthesis; ETR – electron transport rate.

For the leaves of both tree species, the efficiency of photosynthesis Y(II) decreased from spring to autumn, as opposed to chlorophyll fluorescence F/Fm′, whose value in the period under examination increased and reached the maximum value in autumn (Table 4). A reduction by over 54% of photosynthetic efficiency Y(II) of maple leaves in autumn as compared to spring was also observed, which was closely related to chlorophyll *a* content (Table 3). The Y(II) value of lime leaves was decreased by over 35% in autumn as compared to spring. Thus, the highest quantum efficiency of photochemical reaction Y(II) was observed in both tree species in spring and amounted to 0.663 for lime leaves and 0.715 for maple leaves respectively (Table 4).

## Discussion

The investigation conducted by Kitajima^55^ shows that cellulose content and lamina density jointly improve leaf strength, and these carbon-based physical traits, rather than phenolic-based defence, clarify species distinction in herbivory, leaf lifespan and habitat factors. Tensile experiments provided the evidence of variation across tree species and seasons of the year in leaf mechanical properties. Our measurements showed that the most stiffness leaves (the highest values of Young’s modulus) for both species were in the summer. However, late in the senescence process (exactly in autumn) more notable decrease in *E* values was observed in the case of lime leaves. During the study period maple leaves were considerably stiffer than lime leaves (Fig. 1a, Tables 1 and 2). As expected the leaf stiffness is strongly depended on tree species. The major difficulty in comparing all our results of the mechanical properties with other reports, was the lack of scientific studies presenting the values of various strength parameters, e.g. Young’s modulus or critical surface tension of maple and lime leaves. The comparison of the results was only possible in regard to the leaves of other plant species (trees, shrubs) subjected to the tensile force. For oak species, Kawai and Okada^20^ obtained the *E* value of approx. 29 MPa for *Quercus crispula* leaves and approx. 44 MPa for *Quercus serrata* leaves. Méndez-Alonzo^19^ designated Young’s modulus for individual leaf structures of 27 species of shrubs growing in California (USA). As far as whole leaves were concerned, the *E* value obtained in these studies was within the range of 0.64–18.7 MPa.

In general, the range of values of critical surface tension (*σ*_*k*_) for the tested leaves was under 1,6 MPa (Tables 1 and 2). Similar to the leaf stiffness these values depended on tree species. In comparison, the critical surface tension value, as measured by Caldwell^32^ for the leaves of 20 species of shrubs and small trees representing 10 botanical families, ranged from approx. 1 MPa to over 15 MPa for *Pultenaea muelleri* and *Hakea ulicina* leaves. According to Onoda^29^, the average critical surface tension value for over 1000 plant species was approximated 3.31 MPa. With such a large number of leaves of different plant species, it was obvious that there were huge differences of up to 95 times between the highest and the lowest value of the specific mechanical trait. The average critical surface tension value determined by Kawai and Okada^20^ for the leaves of 8 tree species belonging to the beech family (*Fagaceae*) amounted to 2.61 MPa.

For the most shrubs and small tree leaves studied by Caldwell^32^ the force to tear assumed the tenths of N · mm^−1^, with the exception of *Pultenaea muelleri* leaves, for which *f*_*t*_ exceeded 2 N · mm^−1^, and *Hakea ulicina* leaves with the value of over 6 N · mm^−1^. In the studies conducted by Onoda^29^ the average force of leaves tearing for over 900 plant species amounted to 0.87 N · mm^−1^. Depending on the oak species, Kawai and Okada^20^ determined value of the tearing force of the leaf samples within the range of 0.29 N · mm^−1^– 0.34 N · mm^−1^.

Our knowledge of the leaf mechanical properties remains incomplete if we do not examine the values of the Poisson ratio. Using the tensile test and our authorial method - the random markers method^47^ we were able to determine the *v* values (Tables 1 and 2). Due to the technical obstacles encountered by researchers, there is no available literature concerning the *v* value of leaves to compare our results.

The examinations of overall physiological changes - photosynthetic traits, occurred throughout senescence illustrate differences between the tree species (Table 3). For both maple and lime leaves the greenness index has the highest values in the summer. That reflects the interdependence of nitrogen nutritional status of plants. Those results agree with the studies conducted by Fini^58^ where the chlorophyll content obtained from the SPAD-502 meter varied different lime and maple species. According to Wolf^59^ in the process of leaf senescence, the decrease in the chlorophyll *a* concentration is clearly observed, while the chlorophyll *b* content is only slightly altered. Whereas, Koike^6^, showed that both parameters remain stable throughout the growing season of trees. Moy *et al*.^60^ indicate a relatively constant total chlorophyll content in the sugar maple tree leaves (*Acer saccharum* Marsh.), from mid-August to mid-September. Junker and Ensminger^8^ analyzed the concentration of the photosynthetic dyes in the sugar maple green leaves collected in the summer, as well as in the colorful autumn one. The different leaves dyeing also indicated the consecutive stages of their senescing process^8,61^.

In comparison to the green summer leaves the autumn ones contained over four times less total chlorophyll, while for the colorful once only trace amounts of this pigment. Moreover, Koike^6^ revealed that the content of total chlorophyll and *b* fraction in e.g. maple and lime leaves decrease in relation to the height place of the leaves collection in the tree crown (i.e. tree’s vertical profile).

Our measurements of the content of carotenoids showed the lack of differences in the amount of this chlorophyll dyes in examined leaves (Table 3). Lichtenthaler’s study^62^ study concerning chlorophyll and carotenoids content of lime leaves showed that more of these pigments are found in leaves exposed to the sun than in those shaded ones. In the case of the sugar maple, dyes concentration increased from spring to mid-summer, followed by a significant decrease thereafter.

According to Angelini^63^ providing appropriate growth conditions to plants guarantees the photosynthetic efficiency Y(II) of 0.85. Whereas very low values (0.2–0.3) indicate irreversible changes in the PSII structure resulting from the occurrence of stress-inducing factors. Ferrini^64^ highlighted that the trees from urban plantings are exposed to drought, hot air and relatively high soil temperature, as well as low fertility and soil compaction. The salts used in the winter on pavements and streets penetrate into the ground and generate unfavorable environmental conditions for the proper growth. In addition, the damage of the leaves surface of trees growing near the roadway can lead the dysfunction of their photosynthetic apparatus^11,65^.

A study by Lichtenthaler^66^ showed that the average fluorescence value F/Fm′ in the leaves of different tree species (incl. lime and maple) ranged from 0.81 to 0.83 under exposition to the sun and from 0.74 to 0.76 for the shade. The chlorophyll fluorescence in the maple leaves remained at a very high level from mid-August to early October^60^ then there significant reduction in F/Fm′ to the extremely low level in the second decade of October occurred. However, our results indicate that in the case of the analyzed leaves, the F/Fm′ chlorophyll fluorescence value was lower and it ranged from 0.285 (spring) to 0.674 (autumn) for maple leaves, and 0.337 (spring) and 0.572 (autumn) for lime leaves (Table 4).

## Conclusions

Our results revealed that both the season of the year and the tree species strongly influenced the values of strength parameters such as *E*, *σ*_*k*_, *f*_*t*_, and photosynthetic dyes content. The leaves mechanical and phenotype parameters undoubtedly affect the improvement of defenses against herbivores and resistance to environmental factors. Significantly higher values of Young’s modulus, critical surface tension, the force to tear and pigment concentration were determined for the leaves of the maple tree as compared to the lime tree. During the final leaf stage (autumn) the values of the strength parameters were at the intermediate level between the minimum (spring) and the maximum (summer). For the leaves of both tree species, the highest index of photosynthetic efficiency was determined in spring, whereas in summer the leaf greenness index was the highest. Quantum efficiency of photochemical reaction was highest in spring when trees and their leaves needed the most light to grow and develop, while its lowest value was noted in autumn, which was related to the senescing process of leaves.

In order to understand the physical properties affecting leaf lifespan, we propose to extend the existing studies based on mechanical and phenotypic parameters by those, which simultaneously analyze leaf biochemistry. Other studies have stated that by carrying out biochemical analysis, we would be able to determine carbon-based traits e.g. lamina density, which affects the strength and leaf lifespan.

## Material and Methods

In laboratory tests we used the leaves of the two tree species - *Acer platanoides* L. and *Tilia cordata* Mill. growing in close proximity to one another and coming from a private garden (51°22′N, 21°46′E) located in the Masovian Voivodship ~16 km west of Puławy.

**Figure 4:**
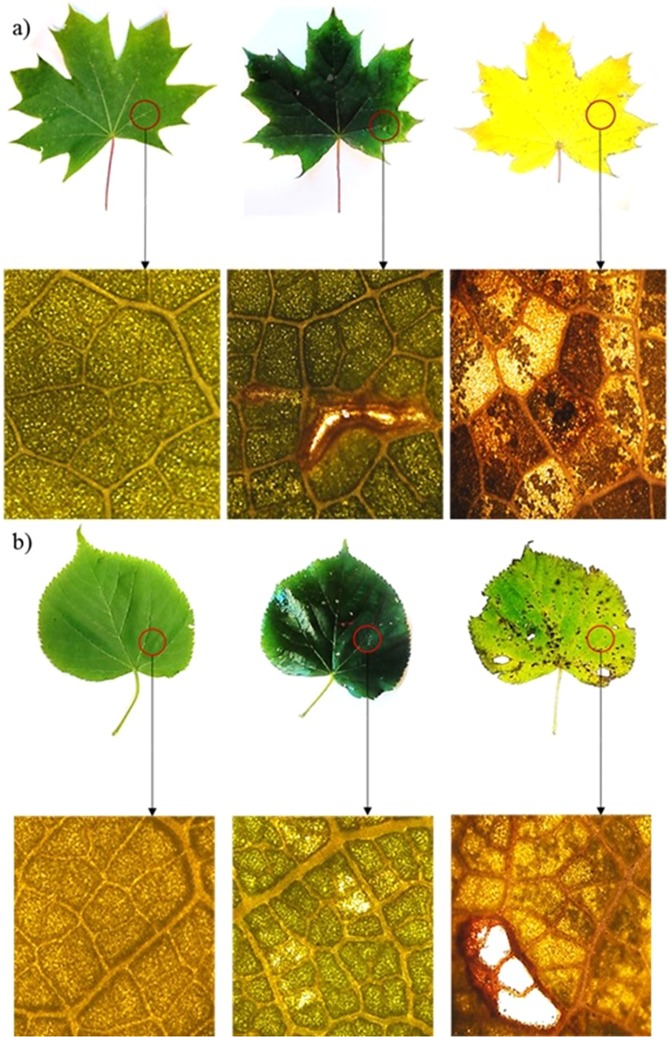
Pictures of green (spring, summer) and yellow/green-brown (autumn) leaves of *Acer platanoides* L. (**a**) and *Tilia cordata* Mill. (**b**) and leaf surface images (quintuplicated) obtained by means of the Leica DM 2500 optical microscope.

Leaf flushing began in the 3rd and 4th week of April for the maple and the lime tree, respectively. The beginning of the research was established at the moment when the leaves reached the shape and size characteristic for the species, i.e. in the 2nd week of May. The tests of leaf mechanical properties and phenotype parameters were conducted during one season phenological observation in 2016, i.e. in mid-May (spring in Poland), mid-August (summer) and mid-October (autumn). The leaves were randomly selected and cut off very carefully so as not to damage their surface. The plant material examined in the middle of May had no visible damage, while in summer and in autumn there were naturally occurring surface damage processes caused by e.g. herbivorous pests and abiotic factors (Fig. 4). The research was completed in mid-October due to the intense fall of leaves in the second half of the month, as a result of which most of the trees were leafless at the beginning of November. The leaves used in the study were evenly sized. The collected plant material was placed in a plastic box lined with damp tissue paper and immediately transported to the laboratory for tension measurement and photosynthetic pigment determination.

The strength tests were conducted on a test-bench equipped with a strain-gauge table and a computer with appropriate software to determine the value of Young’s modulus and Poisson’s ratio on the basis of an authorial method^47^ based on image analysis as well as the analysis of the positions of points randomly placed upon the material under examination (random markers method)^47,67^. The main advantages of applying this method are, among others, the possibility of omitting boundary conditions, thus making the obtained test results independent from the deformations of the material occurring in the area of critical sections^68^, as well as determining the precise correlation between the tensile force and the degree of sample deformation^47^. It cannot be ignored that the measurements can be made anywhere in the material under examination, even if it is partially damaged. An additional advantage is the possibility of observing the natural course of sample deformation as a result of the tensile force^47^. Young’s modulus, Poisson’s ratio and critical surface tension of the leaves under examination were determined on the basis of a single-axial tensile test. The samples for laboratory tests were shaped like dumbbells of 50 mm in length and 20 mm in width and were cut out by means of a specially designed blanking die so that the main leaf vein was located on the symmetry axis of the dumbbells. The method in question requires the provision of a third dimension (thickness) of the sample cut out for examination. The measurement of this size was done in 3 points: 4 times on the main nerve of the leaf, the lateral nerves and on the leaf blade itself by means of a micrometer screw accurate to within 0.01 mm. Sample thickness was the average value of its 12 unitary measurements. The sample was fastened to the terminal of a universal testing machine and then stretched at the rate of force accretion *dF*/*dt* of 0.13 N · s^−1^. The starting point of the measurement was preceded by depositing powdered graphite markers. The image of the stretched sample surface along with its markers was transferred from the camera to the memory of the computer analyzing the changes in the distance between the points during the stretching process^47^. Each measuring series consisted of 30 repetitions. Young’s modulus *E* for each sample was determined on the basis of the direction quotient of a straight line approximating a single dependence *ε*_*x*_ = *f(σ)*, where *ɛ*_*x*_ is the relative elongation in the direction of the x-axis, and *σ* is tension (MPa). Critical surface tension σ_*k*_ (MPa) was determined on the basis of relation (1), Poisson’s ratio *v* (−) according to (2), and the force to tear *f*_*t*_ (N · mm^−1^) according to formula (3):

1 $${\sigma }_{k}=\frac{{F}_{{\max }}}{S}$$

2 $$v=-\,\frac{{\varepsilon }_{y}}{{\varepsilon }_{x}}$$

3 $${f}_{t}=\frac{{F}_{{\max }}}{w}$$

where:

*F*_*max*_ - maximum force at which the sample is destroyed (N),

*S* - sample cross-sectional area (mm^2^),

*ε*_*x*_ - relative elongation of the sample in the direction of the acting force *F*,

*ε*_*y*_ - relative deformation of the sample in the direction perpendicular to the applied force *F*,

*w* - sample width (mm).

Collecting data during the measurement and doing necessary calculations in order to determine the mechanical parameters were controlled by the computer program “Videoo”^67^. The total value of the determined strength quantities was the average of all the unitary measurements.

The determination of photosynthetic pigments (chlorophyll *a*, chlorophyll *b* and carotenoids) was performed by means of the spectrophotometric method in three repetitions for each leaf. As for measuring the leaf greenness index by using the chlorophyll marker SPAD-502 and testing chlorophyll fluorescence, quantum efficiency of photosynthesis Y(II) and the rate of electron transport through photosystems (ETR) by means of the Mini-PAM 2000 by Waltz Germany, the repetitions were conducted ten times on each leaf.

Photosynthetic pigments were determined by two methods. In the first one, chlorophyll and carotenoids were defined by isolating them from the leaves in the dark, using acetone containing 0.01% w/v BHT (butylated hydroxytoluene) so as to prevent oxidation. The UV-Vis spectra were measured by means of a Carry Bio 300 dual-beam spectrophotometer, while pigment concentrations were calculated on the basis of Lichtenthaler and Buschmann’s procedure^69^. In the second method, the chlorophyll marker SPAD-502 was used to receive the result automatically, without the necessity to collect and destroy the samples. The procedure involved grasping the leaf into a measurement clip equipped on one side with the source of light and a photodetector measuring the amount of light passing through the leaf on the other. The pressure of the clip caused overexposure of the sample, while the photodetector, measuring the radiation of 650 nm in wavelength, made it possible to determine the amount absorbed by chlorophyll. In order to correct the result calculated by the microprocessor and expressed in conventional units on the display, 940 nm-wavelength light absorbed by the rest of the structure was used. After two seconds another measurement was possible^70^.

Quantum efficiency of photochemical reaction Y(II) (the relation between the quantum used in photochemical transmutation and the total amount of photosynthetically active radiation PAR) and electron transport rate ETR^11^ were measured by means of modulated fluorescence (PAM). The method involved measuring the chlorophyll fluorescence signal by means of the Mini-PAM 200 Photosynthesis Field analyzer by Waltz Germany. In the process of emitting modulated light pulses (switched on and off at high frequencies) onto the photosynthetic sample, the fluorescence signal of agitated chlorophyll *a* molecules (PS II) was recorded^71,72^. The analysis of the results was conducted by means of WinControl Software for PAM Fluorometers (Waltz Germany).

The datasets generated during and/or analysed during the current study are available from the corresponding author on reasonable request.
